# A Machine Learning–Based Prediction Model for Acute Kidney Injury in Patients With Community-Acquired Pneumonia: Multicenter Validation Study

**DOI:** 10.2196/51255

**Published:** 2024-12-19

**Authors:** Mengqing Ma, Caimei Chen, Dawei Chen, Hao Zhang, Xia Du, Qing Sun, Li Fan, Huiping Kong, Xueting Chen, Changchun Cao, Xin Wan

**Affiliations:** 1 Department of Nephrology Nanjing First Hospital, Nanjing Medical University Nanjing China; 2 Department of Nephrology Wuxi People's Hospital Affiliated with Nanjing Medical University Wuxi China; 3 Department of Nephrology Sir Run Run Hospital, Nanjing Medical University Nanjing China; 4 Department of Nephrology Xinyi people's Hospital Xuzhou China

**Keywords:** acute kidney injury, community-acquired, pneumonia, machine learning, prediction model

## Abstract

**Background:**

Acute kidney injury (AKI) is common in patients with community-acquired pneumonia (CAP) and is associated with increased morbidity and mortality.

**Objective:**

This study aimed to establish and validate predictive models for AKI in hospitalized patients with CAP based on machine learning algorithms.

**Methods:**

We trained and externally validated 5 machine learning algorithms, including logistic regression, support vector machine, random forest, extreme gradient boosting, and deep forest (DF). Feature selection was conducted using the sliding window forward feature selection technique. Shapley additive explanations and local interpretable model-agnostic explanation techniques were applied to the optimal model for visual interpretation.

**Results:**

A total of 6371 patients with CAP met the inclusion criteria. The development of CAP-associated AKI (CAP-AKI) was recognized in 1006 (15.8%) patients. The 11 selected indicators were sex, temperature, breathing rate, diastolic blood pressure, C-reactive protein, albumin, white blood cell, hemoglobin, platelet, blood urea nitrogen, and neutrophil count. The DF model achieved the best area under the receiver operating characteristic curve (AUC) and accuracy in the internal (AUC=0.89, accuracy=0.90) and external validation sets (AUC=0.87, accuracy=0.83). Furthermore, the DF model had the best calibration among all models. In addition, a web-based prediction platform was developed to predict CAP-AKI.

**Conclusions:**

The model described in this study is the first multicenter-validated AKI prediction model that accurately predicts CAP-AKI during hospitalization. The web-based prediction platform embedded with the DF model serves as a user-friendly tool for early identification of high-risk patients.

## Introduction

Acute kidney injury (AKI) is common in patients with community-acquired pneumonia (CAP), which is associated with higher morbidity and mortality, longer hospital stays, and increased financial costs. In recent years, outbreaks of pneumonia caused by new pathogens, including SARS in 2002, Middle East respiratory syndrome in 2012, and the COVID-19 pandemic in 2019, have severely strained resources and had significant societal impacts. In addition, a considerable percentage of patients with CAP, ranging from 4.3% (429/10,069) to 34.4% (631/1836), develop AKI [1-3]. Identifying and intervening in AKI early can facilitate the timely restoration of renal function, ultimately enhancing survival rates and aiding patients in their recovery post discharge [4,5]. However, AKI is primarily detected through elevated levels of serum creatinine (Scr), which is a delayed indicator of AKI. Although various novel urine and plasma biomarkers have shown promising results in diagnosing AKI, only a few are presently used in clinical practice [6]. Furthermore, the cost associated with biomarker assessment and the challenges in interpreting the results have limited their widespread adoption.

In recent years, machine learning (ML) techniques have offered the potential to develop accurate prediction models for AKI, enabling early risk stratification and personalized interventions. A retrospective study published in *Nature* in 2019 included a total of 703,782 adult patients from 172 hospitals and 1062 outpatient facilities. By using the recurrent neural network algorithm, this study demonstrated the ability to predict 55.8% (18,837/33,759) of patients with AKI 48 hours in advance, with an impressive area under the receiver operating characteristic curve (AUC) of 92% [7]. These findings suggest that ML can accurately predict the occurrence of AKI at an early stage.

Numerous studies have focused on ML models for predicting AKI in patients. However, these models have primarily focused on vigorous patients, patients with sepsis, patients with cardiac surgery, patients with cancer, and other specific populations. Currently, there is a lack of models for predicting AKI in patients with CAP. Furthermore, most of the studies have relied on databases like MIMIC-III (The Medical Information Mart for Intensive Care III), with a scarcity of local patient databases and a lack of external validation. Therefore, there is an urgent need to develop AKI prediction models specifically tailored to the population of patients with CAP to provide targeted guidance for diagnosis and treatment. This study aimed to use ML methods to develop models that accurately predict CAP-associated AKI (CAP-AKI).

## Methods

### Study Population

This retrospective cohort study involved 3 independent tertiary hospitals—Wuxi People’s Hospital, Nanjing First Hospital, and Sir Run Run Hospital affiliated with Nanjing Medical University. Data were collected from January 2016 to December 2017. The inclusion criteria included patients aged 18 years and older who were diagnosed with CAP during hospitalization. The study exclusion criteria were (1) patients diagnosed with stage 4-5 chronic kidney disease (CKD) or receiving regular renal replacement therapy, (2) patients with a history of kidney transplant, (3) patients with a hospital stay of less than 24 hours or exceeding 90 days, (4) patients who had been treated by other medical institutions within 14 days before hospitalization, (5) patients who relied on mechanical ventilation and palliative care for advanced tumors on a chronic basis, (6) occurrence of AKI within 24 hours of admission or 7 days thereafter, and (7) patients with peak Scr levels less than 53 μmol/L or Scr measurements fewer than 2 times between 1 year before hospitalization and discharge or fewer than 1 time during hospitalization.

### Primary Outcome

The primary outcome for developing the ML models was AKI in patients with CAP. AKI is identified based on the Scr level, as stated by the Kidney Disease Improving Global Outcomes (KDIGO) [8]. It is defined as an increase in Scr to ≥1.5 times baseline within 7 days of admission, or an increase in Scr ≥0.3 mg/dL within 48 hours. The baseline Scr level was determined as the average within 7 to 365 days before hospitalization. If baseline Scr is not recorded, the first Scr value upon admission is used as the baseline Scr. Urine output was not considered for AKI diagnosis due to missing data [9]. Estimated glomerular filtration rate (eGFR) was calculated using the Chronic Kidney Disease Epidemiology Collaboration (CKD-EPI) creatinine equation [10]. Proteinuria is defined as “The presence of at least 1+ protein in urinalysis.”

### Training and Validation Data

The dataset was divided into 2 parts: one from Wuxi People’s Hospital for training and internal validation (2568/3211, 80% for model development and 643/3211, 20% for validation) and the other from the multicenter patients for external validation.

### Data Processing

Baseline characteristics and clinical data of patients within 24 hours of admission, including 62 indicators, were obtained from the electronic record system. Variables with over 15% (9/62) missing values were excluded, and the remaining variables were imputed using the missForest method. Outliers were identified and treated as missing values. The data of each feature were standardized using the *z* score method. Variables with strong collinearity (variance inflation factor [VIF] value≥10) were discarded using the VIF. The synthetic minority over-sampling technique (SMOTE) was applied to address data imbalance and improve the prediction efficiency of the models.

### Feature Selection and Machine Learning Models Establishment

Feature importance was evaluated using the *sklearn* library method. Features were sorted in descending order based on their scores. A sliding windows sequential forward feature selection technique method based on random forest (RF) and out-of-bag (OOB) filtering was used to select features related to AKI. The optimal number of features is determined by finding the subset that results in the lowest OOB error (or the highest OOB score). After feature selection, 5 ML algorithms, including logistic regression (LR), RF, support vector machine, extreme gradient boosting (XGBoost), and deep forest (DF) models, were used to construct the prediction models. A detailed description of ML methods was given in Multimedia Appendix 1.

### Model Validation and Performance

External datasets were used to validate the models, and their performances were evaluated using various metrics such as the AUC, specificity, sensitivity, positive predictive value (PPV), negative predictive value, *F*_1_-score, expected calibration error (ECE), calibration curve, and decision curve. A 1000-bootstrap method was used to determine CIs by sampling with replacement on the prediction indices.

### Explanation of the Model

Shapley additive explanations (SHAP) is a method that constructs an additive interpretation model. It considers all features as “contributors” and calculates the marginal contribution of each feature when added to the model output. SHAP aims to provide explanations for “black box models” at both global and local levels, making the model more understandable and applicable.

On the other hand, local interpretable model-agnostic explanations (LIME) modify a single data sample by adjusting the eigenvalues and observing its effect on the output. LIME acts as an “interpreter” by explaining the prediction of each data sample. In LIME, a set of interpretations represents the contribution of each feature to the prediction of a single sample.

### Statistical Analysis

To compare baseline characteristics across different datasets, we used 1-way ANOVA and chi-square tests. Statistical significance was determined using a 2-sided *P* value of less than .05. The ML models were created using Python (version 3.9.7; Python Software Foundation) with packages such as *scikit-learn*, *NumPy*, *SHAP*, and *pandas*, as well as R (version 4.1.0; R Core Team) with the *glmnet* package.

### Ethical Considerations

The study was approved by the institutional review boards of Wuxi People’s Hospital (KY22073), Sir Run Run Hospital (2021-SR-042), and Nanjing First Hospital (KY20230410-01-KS-01). The study was conducted in accordance with the declaration of Helsinki. Due to its retrospective design, the ethics committees from Wuxi People’s Hospital, Sir Run Run Hospital, and Nanjing First Hospital waived the need for informed consent. Data can be analyzed secondary without additional consent. The personal information of patients was kept confidential throughout the entire study. Since the experimental design did not involve any intervention for the patients, no compensation was paid to the patients in the study.

## Results

### Baseline Characteristics

**Figure 1:**
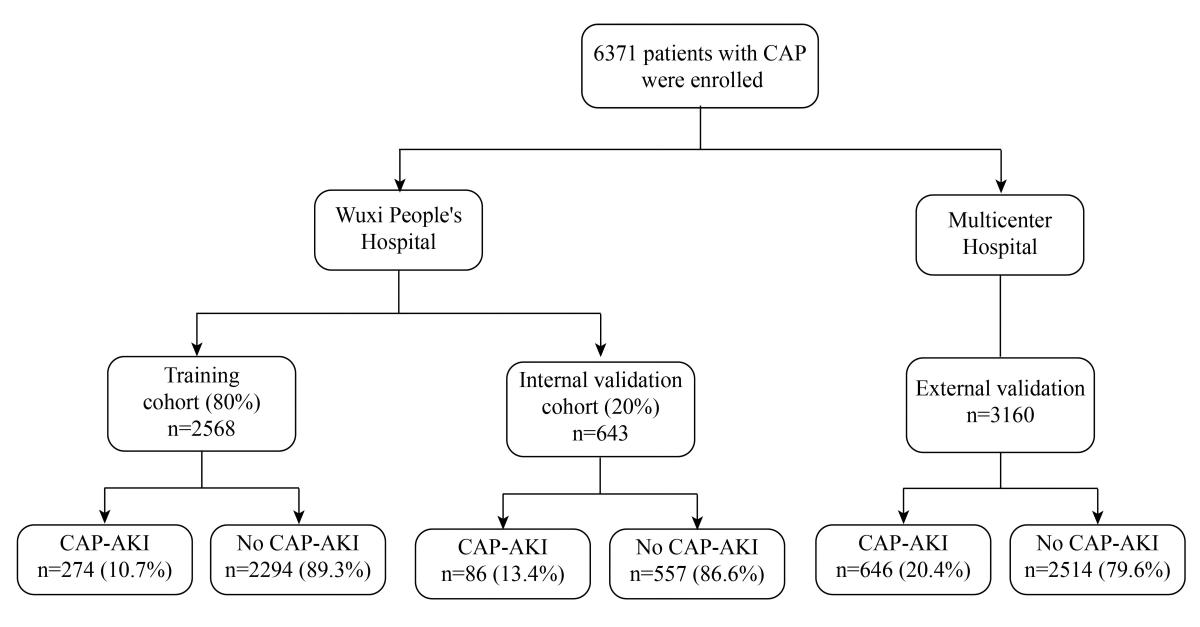
Flowchart for patient selection. CAP: community-acquired pneumonia; CAP-AKI: community-acquired pneumonia–associated acute kidney injury.

The study comprised a total of 6371 patients, with 3211 (50.4%) patients assigned to the training and internal validation groups, and 3160 (49.6%) patients assigned to the external validation group (Figure 1). Out of the total patients, 2566 (40.2%) were female, with a mean age of 67.0 (SD 17.6) years and a baseline Scr level of 72.5 (SD 31.1) μmol/L. Table 1 illustrates the differences between the training and validation sets.

**Table 1:** Baseline characteristics of the patients with community-acquired pneumonia.

Variables	All (n=6371)	Training set (n=2568)	Internal validation set (n=643)	External validation set (n=3160)	*P* value
Female, n (%)	2566 (40.2)	996 (38.8)	227 (35.3)	1343 (42.5)	<.001
Age (years), mean (SD)	67.0 (17.6)	65.0 (17.9)	65.7 (17.2)	68.8 (17.3)	<.001
Hypertension, n (%)	3007 (47.1)	1227 (47.8)	296 (46)	1484 (46.9)	.68
Diabetes, n (%)	1416 (22.2)	720 (28)	201 (31.3)	495 (15.6)	<.001
CKD^a^, n (%)	420 (6.6)	209 (8.1)	61 (9.5)	150 (4.7)	<.001
CVD^b^, n (%)	1322 (20.7)	484 (18.8)	127 (19.8)	711 (22.5)	.003
Cerebrovascular disease, n (%)	977 (15.3)	379 (14.7)	94 (14.6)	504 (15.9)	.40
Chronic lung disease, n (%)	1965 (30.8)	774 (30.1)	195 (30.3)	996 (31.5)	.51
Cancer, n (%)	469 (7.4)	205 (8)	49 (7.6)	215 (6.8)	.23
Mechanical ventilation, n (%)	345 (5.4)	127 (4.9)	30 (4.7)	188 (5.9)	.17
Temperature^c^ (℃), mean (SD)	37.3 (1.2)	37.8 (1.2)	37.8 (1.2)	36.9 (1.8)	<.001
Heart rate^d^ (beats/minute), mean (SD)	84.3 (14.9)	83.7 (14.2)	83.6 (14.2)	85.0 (15.6)	.001
Breathing rate^e^ (births/minute), mean (SD)	19.6 (4.0)	19.5 (3.7)	19.3 (3.1)	19.7 (4.3)	.02
Systolic BP^f,g^ (mm Hg), mean (SD)	126.6 (18.3)	127.6 (19.7)	126.9 (19.5)	125.7 (16.4)	<.001
Diastolic BP^h^ (mm Hg), mean (SD)	76.1 (10.9)	77.4 (19.3)	76.9 (10.7)	75.1 (11)	<.001
Acute respiratory failure, n (%)	598 (9.4)	320 (12.5)	79 (12.3)	199 (6.2)	<.001
Acute cardiac dysfunction, n (%)	354 (5.6)	200 (7.8)	55 (8.6)	99 (3.1)	<.001
ACEI^i^ or ARB^j^, n (%)	910 (14.3)	369 (14.4)	93 (14.5)	447 (14.1)	.97
Diuretic, n (%)	1452 (22.7)	414 (16.1)	110 (17.1)	928 (29.3)	<.001
CCB^k^, n (%)	1251 (19.6)	546 (21.2)	129 (20)	576 (18.2)	.02
β-block, n (%)	364 (5.7)	149 (5.8)	40 (6.2)	175 (5.5)	.77
Baseline Scr^l^ (μmol/L), mean (SD)	72.5 (31.1)	72.5 (22.5)	73.7 (24.3)	72.2 (37.3)	.52
eGFR^m^ (mL/min×1.73m^2^), mean (SD)	84.9 (27.6)	84.3 (25.7)	82.8 (25.4)	85.8 (29.3)	.02
WBC^n^ (×10^9^/L), median (IQR)	6.9 (5.2-9.5)	6.9 (5.3-9.5)	7.0 (5.2-9.5)	6.4 (4.9-8.8)	.03
Neutrophil count (×10^9^/L), median (IQR)	4.9 (3.4-7.4)	4.8 (3.3-7.4)	4.8 (3.4-7.1)	5.0 (3.5-7.6)	.004
Platelet (×10^9^/L), mean (SD)	210.0 (87.9)	211.7 (86.0)	214.9 (107.4)	208.1 (88.4)	.22
CRP^o^ (mg/L), median (IQR)	21.9 (4.0-74.2)	14.0 (2.0-48.9)	13.9 (2.0-48.3)	19.1 (6.4-73.9)	<.001
Hemoglobin (g/L), mean (SD)	121.1 (20.0)	123.0 (18.7)	122.6 (19.2)	119.3 (20.9)	<.001
Albumin (g/L), mean (SD)	32.9 (5.7)	32.0 (5.9)	31.7 (5.9)	33.9 (5.3)	<.001
BUN^p^ (mmol/L), median (IQR)	5.1 (3.7-4.7)	5.0 (3.5-6.3)	4.9 (3.2-6.2)	5.4 (4.3-8.0)	<.001
Potassium (mmol/L), mean (SD)	3.8 (0.5)	3.8 (0.4)	3.8 (0.5)	3.7 (0.5)	<.001
Natrium (mmol/L), mean (SD)	138.8 (5.1)	138.8 (3.7)	138.5 (3.9)	138.9 (6.0)	.08
Chloride (mmol/L), mean (SD)	101.9 (5.6)	101.5 (4.6)	101.2 (5.3)	102.4 (6.0)	<.001
Proteinuria^q^, n (%)	1927 (30.2)	942 (36.7)	239 (37.2)	746 (23.6)	<.001

^a^CKD: chronic kidney disease.

^b^CVD: cardiovascular disease.

^c^The peak temperature on the day of admission.

^d^The immediate admission heart rate.

^e^The immediate admission respiratory rate, or if mechanical ventilation has been initiated, select the respiratory rate upon emergency department admission.

^f^BP: blood pressure.

^g^The systolic blood pressure at admission, or if vasopressor medication has been administered, select the systolic blood pressure upon emergency department admission.

^h^the diastolic blood pressure at admission, or if vasopressor medication has been administered, select the diastolic blood pressure upon emergency department admission.

^i^ACEI: angiotensin-converting enzyme inhibitor.

^j^ARB: angiotensin receptor antagonists.

^k^CCB: calcium-channel blockers.

^l^Scr: serum creatinine.

^m^eGFR: estimated glomerular filtration rate.

^n^WBC: white blood cell.

^o^CRP: C-reactive protein.

^p^BUN: blood urea nitrogen.

^q^Proteinuria: positive urine protein.

Among the 6371 patients, 1006 (15.8%) developed AKI, with the median time for AKI diagnosis being day 3 (IQR 2-4) post admission. A comparison between patients with and without AKI is presented in Table 2. Patients with AKI exhibited higher age, temperature, heart rate, breathing rate, and lower diastolic blood pressure levels. They also had a higher incidence of hypertension, CKD, cardiovascular disease, cerebrovascular diseases, acute respiratory failure, and acute cardiac dysfunction and a higher rate of mechanical ventilation compared with those without AKI. Furthermore, patients with CAP having AKI demonstrated significantly elevated baseline Scr, white blood cell count, neutrophil count, C-reactive protein (CRP), and blood urea nitrogen (BUN) levels compared with those without AKI (all *P*<.05). Conversely, baseline eGFR, platelet count, hemoglobin, and blood albumin levels were substantially lower in the non-AKI group (all *P*<.05).

**Table 2:** Clinical features of patients in the AKI^a^ and non-AKI groups.

Variables	All (n=6371)	AKI group (n=1006)	Non-AKI group (n=5365)	*P* value
Female, n (%)	2566 (40.2)	287 (28.5)	2279 (42.4)	<.001
Age (years), mean (SD)	67.0 (17.6)	77.3 (13.6)	65.1 (17.7)	<.001
Hypertension, n (%)	3007 (47.1)	617 (61.3)	2390 (44.5)	<.001
Diabetes, n (%)	1416 (22.2)	200 (19.8)	1216 (22.6)	.05
CKD^b^, n (%)	420 (6.6)	213 (21.2)	207 (3.9)	<.001
CVD^c^, n (%)	1322 (20.7)	347 (34.5)	975 (18.2)	<.001
Cerebrovascular disease, n (%)	977 (15.3)	192 (19.1)	785 (14.6)	<.001
Chronic lung disease, n (%)	1965 (30.8)	305 (30.3)	1660 (30.9)	.71
Cancer, n (%)	469 (7.4)	76 (7.5)	393 (7.3)	.79
Mechanical ventilation, n (%)	345 (5.4)	187 (18.6)	158 (2.9)	<.001
Temperature (℃), mean (SD)	37.8 (1.2)	38.0 (1.2)	37.7 (1.2)	.002
Heart Rate (beats/minute), mean (SD)	84.3 (14.9)	89.9 (18.8)	83.3 (13.9)	<.001
Breathing rate (births/minute), mean (SD)	19.6 (4.0)	21.6 (5.3)	19.2 (3.6)	<.001
Systolic BP^d^ (mm Hg), mean (SD)	126.6 (18.3)	126.4 (19.7)	126.6 (18.0)	.71
Diastolic BP (mm Hg), mean (SD)	76.1 (10.9)	72.4 (13.7)	76.8 (10.2)	<.001
Acute respiratory failure, n (%)	598 (9.4)	200 (19.9)	398 (7.4)	<.001
Acute cardiac dysfunction, n (%)	354 (5.6)	125 (12.4)	229 (4.2)	<.001
ACEI^e^ or ARB^f^, n (%)	910 (14.3)	188 (18.7)	722 (13.5)	<.001
Diuretic, n (%)	1452 (22.7)	598 (59.4)	854 (15.9)	<.001
CCB^g^, n (%)	1251 (19.6)	210 (20.8)	1041 (19.4)	.28
β-block, n (%)	364 (5.7)	62 (6.1)	302 (5.6)	.51
Baseline Scr^h^ (μmol/L), mean (SD)	72.5 (31.1)	85.3 (52.1)	70.1 (24.6)	<.001
eGFR^i^ (ml/min×1.73m^2^), mean (SD)	84.9 (27.6)	71.9 (30.7)	87.4 (26.3)	<.001
WBC^j^ (×10^9^/L), median (IQR)	6.9 (5.2-9.5)	8.8 (6.3-12.7)	6.7 (5.1-9.0)	.03
Neutrophil count (×10^9^/L), median (IQR)	4.9 (3.4-7.4)	7.2 (4.9-11.1)	4.6 (3.2-6.8)	.004
Platelet (×10^9^/L), mean (SD)	210.0 (87.9)	180.4 (92.8)	215.5 (84.4)	<.001
CRP^k^ (mg/L), median (IQR)	21.9 (4.0-74.2)	67.6 (18.8-131.0)	17.2 (3.2-57.8)	<.001
Hemoglobin (g/L), mean (SD)	121.1 (20.0)	112.1 (25.0)	122.8 (18.4)	<.001
Albumin (g/L), mean (SD)	32.9 (5.7)	29.5 (5.6)	33.6 (5.5)	<.001
BUN^l^ (mmol/L), median (IQR)	5.1 (3.7-4.7)	9.5 (6.6-14.2)	4.8 (3.6-6.1)	<.001
Potassium (mmol/L), mean (SD)	3.8 (0.5)	3.9 (0.7)	3.7 (0.4)	<.001
Natrium (mmol/L), mean (SD)	138.8 (5.1)	138.9 (7.3)	138.8 (4.6)	.85
Chloride (mmol/L), mean (SD)	101.9 (5.6)	102.0 (5.6)	102.0 (5.1)	.84
Proteinuria, n (%)	1927 (30.2)	536 (53.3)	1391 (25.9)	<.001

^a^AKI: acute kidney injury.

^b^CKD, chronic kidney disease.

^c^CVD: cardiovascular disease.

^d^BP: blood pressure.

^e^ACEI: angiotensin-converting enzyme inhibitor.

^f^ARB: angiotensin receptor antagonists.

^g^CCB: calcium-channel blockers.

^h^Scr: serum creatinine.

^i^eGFR: estimated glomerular filtration rate.

^j^WBC: white blood cell.

^k^CRP: C-reactive protein.

^l^BUN: blood urea nitrogen.

### Model Validation and Performance

In the training model, 9 variables with over 15% (9/62) missing values were excluded from the final cohort. These variables included blood lactic acid, ferritin, glycosylated hemoglobin C, blood oxygen partial pressure, blood carbon dioxide partial pressure, sputum pathogens, complement C3, complement C4, and procalcitonin. Among the remaining 53 variables, 3 had VIF values≥10. These variables were inhaled oxygen concentration, oxygen inhalation status, and vasoactive drug application. After eliminating variables with collinearity, the remaining 50 features (including age, sex, smoking status, and severe pneumonia), comorbidities (including history of chronic lung disease, CKD, malignancy, hypertension, diabetes, cardiovascular disease, chronic liver disease, and cerebrovascular disease), complications (such as septic shock, acute respiratory failure, and acute cardiac dysfunction), vital signs (including temperature, heart rate, respiratory rate, systolic blood pressure, and diastolic blood pressure), treatment on the day of admission (including noninvasive ventilation, mechanical ventilation, antiplatelet therapy, anticoagulation, renin-angiotensin-aldosterone system [RAAS] inhibitors, proton pump inhibitors, statins, diuretics, nonsteroidal anti-inflammatory drugs, insulin, calcium channel blockers, beta-blockers, and corticosteroids), and laboratory tests (including baseline Scr, baseline eGFR, CRP, urine protein, serum albumin, white blood cell count, platelet count, hemoglobin, neutrophil count, monocyte count, lymphocyte count, BUN, alanine aminotransferase [ALT], aspartate aminotransferase [AST], serum potassium, serum chloride, and serum sodium) were evaluated using the sliding windows sequential forward feature selection technique method.

**Figure 2:**
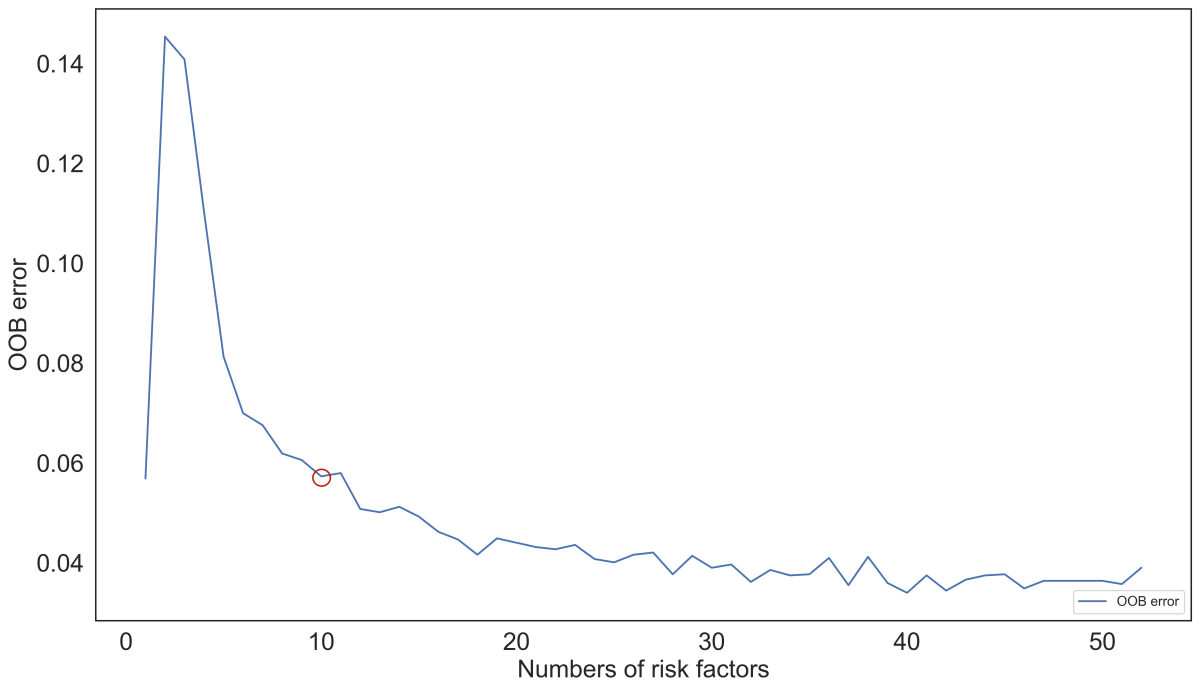
Feature selection based on the sliding windows sequential forward feature selection technique process. The optimal number of features was determined according to the OOB value. A lower OOB value and fewer variables included results in the optimal combination of features (11 at the red circle). OOB: out-of-bag.

**Figure 3:**
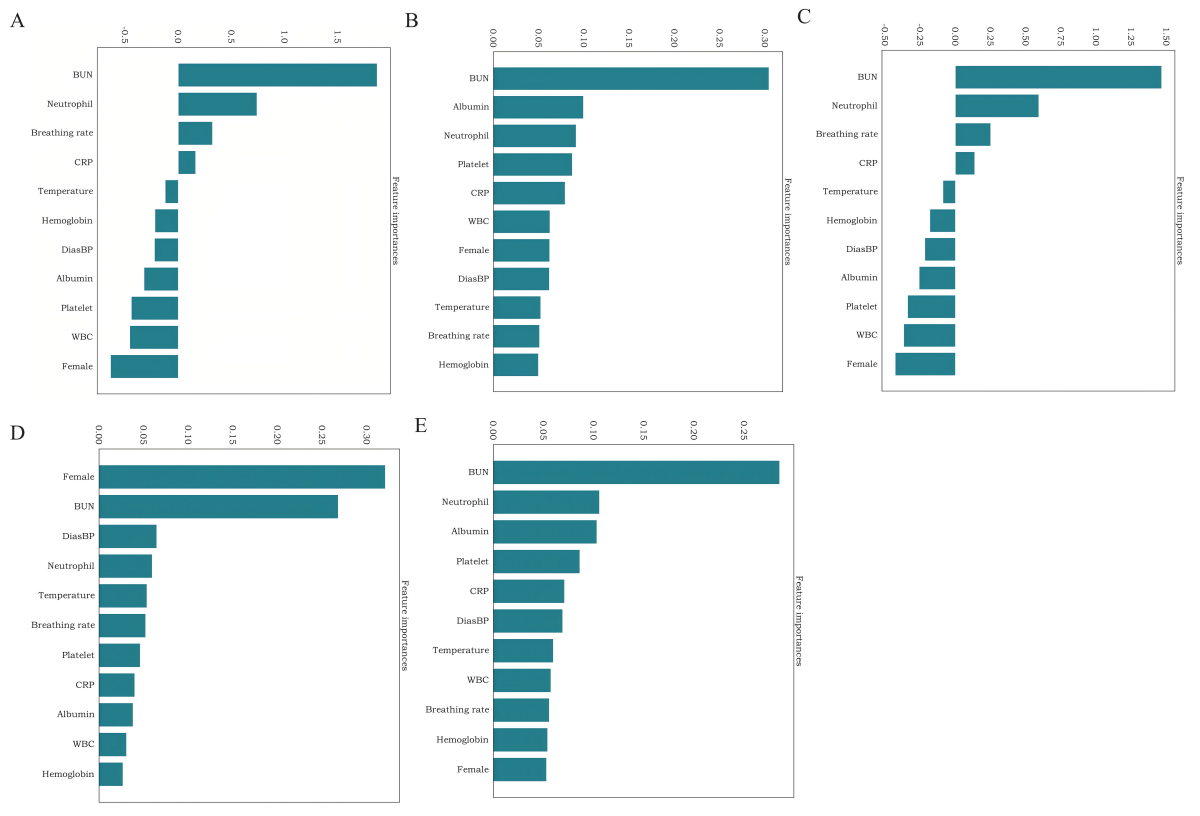
Feature importance derived from the machine learning models. (A) logistic regression model, (B) random forest model, (C) support vector machine model, (D) extreme gradient boosting model, and (E) deep forest model. BUN: blood urea nitrogen; CRP: C-reactive protein; DiasBP: diastolic blood pressure; WBC: white blood cells.

The model generated an OOB value for each included feature. Figure 2 indicates that as the number of features increased to 11, the OOB value (0.579773) decreased substantially, and subsequently, the OOB value remained stable with increasing features. When the number of features reached 40, the OOB value (0.0340017) reached its lowest point. Therefore, to facilitate clinical application, we selected the first 11 features as the final model variables. These variables included sex, temperature, breathing rate, diastolic blood pressure, CRP, albumin, white blood cell count, platelet count, hemoglobin, neutrophil count, and BUN. Figure 3 illustrates that the BUN and neutrophil count consistently ranked among the top 5 indicators in all 5 models, suggesting their significant contribution to the occurrence of AKI.

### Internal Validation of the Model

**Figure 4:**
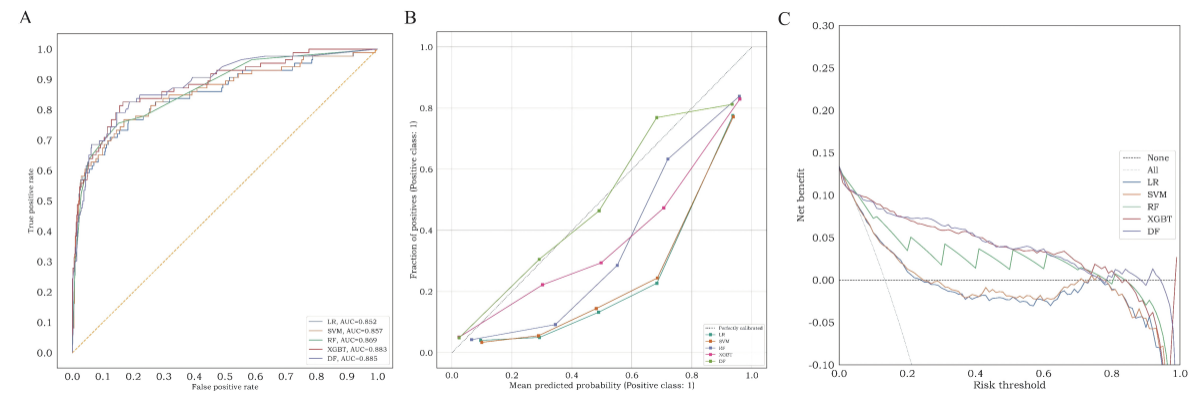
The performance of machine learning models in the internal validation set. (A) area under receiver operating characteristic curve, (B) calibration curve, and (C) decision curve analysis curve. AUC: area under the receiver operating characteristic curve; DF: deep forest; LR: logistic regression; RF: random forest; SVM: support vector machine; XGBT: extreme gradient boosting.

A total of 643 patients were included in the internal validation, with an AKI incidence of 13.5% (n=87). Figure 4A illustrates the AUC of the 5 models in the internal validation set. Among them, the DF model exhibited the highest discrimination compared with the other 4 models, achieving an AUC of 0.89 (95% CI 0.84-0.92). The XGBoost model followed closely with an AUC of 0.88 (95% CI 0.84-0.92). Detailed prediction performance of the 5 ML models is presented in Table 3. The DF model demonstrated the best specificity prediction for AKI (0.99, 95% CI 0.98-0.99). Compared with the other models except DF, the XGBoost model showed higher accuracy (0.92, 95% CI 0.90-0.94), PPV (0.78, 95% CI 0.68-0.86), and *F*_1_-score (0.63, 95% CI 0.54-0.70). Calibration curves of the 5 models are depicted in Figure 4B. The results indicated that the DF model exhibited the lowest ECE value of 0.03, signifying superior calibration. The XGBoost model followed with an ECE of 0.05. Decision curve analysis of the prediction models is presented in Figure 4C. The findings revealed that the DF, XGBoost, and RF models yielded net benefits within the threshold range of 0-0.8. In the internal validation set, the DF model demonstrated the highest predictive ability for AKI in patients with CAP.

**Table 3:** Detailed prediction performance of 5 machine learning models in the internal validation set.

Models	AUC^a^, (95% CI)	Accuracy, (95% CI)	Sensitivity, (95% CI)	Specificity, (95% CI)	PPV^b^, (95% CI)	NPV^c^, (95% CI)	*F*_1_-score, (95% CI)	ECE^d^
LR^e^	0.85 (0.81-0.90)	0.75 (0.72-0.77)	0.81 (0.74-0.88)	0.73 (0.70-0.77)	0.32 (0.27-0.37)	0.96 (0.95-0.98)	0.46 (0.40-0.52)	0.12
SVM^f^	0.86(0.81-0.90)	0.76 (0.72-0.78)	0.79 (0.71-0.87)	0.75 (0.72-0.78)	0.33 (0.28-0.38)	0.96 (0.94-0.97)	0.46 (0.40-0.52)	0.12
RF^g^	0.87 (0.83-0.90)	0.91 (0.89-0.93)	0.52 (0.43-0.61)	0.97 (0.96-0.98)	0.74 (0.65-0.83)	0.93 (0.91-0.95)	0.61 (0.53-0.69)	0.08
XGBoost^h^	0.88 (0.84-0.92)	0.92 (0.90-0.94)	0.52 (0.43-0.61)	0.98 (0.97-0.99)	0.78 (0.68-0.86)	0.93 (0.91-0.95)	0.63 (0.54-0.70)	0.05
DF^i^	0.89 (0.84-0.92)	0.90 (0.88-0.91)	0.31 (0.23-0.39)	0.99 (0.98-0.99)	0.77 (0.65-0.89)	0.90 (0.88-0.92)	0.45 (0.34-0.53)	0.03

^a^AUC: area under the receiver operating characteristic curve.

^b^PPV: positive predictive value.

^c^NPV: negative predictive value.

^d^ECE: expected calibration error.

^e^LR: logistic regression.

^f^SVM: support vector machine.

^g^RF: random forest.

^h^XGBoost: extreme gradient boosting.

^i^DF: deep forest.

### External Validation of the Model

**Figure 5:**
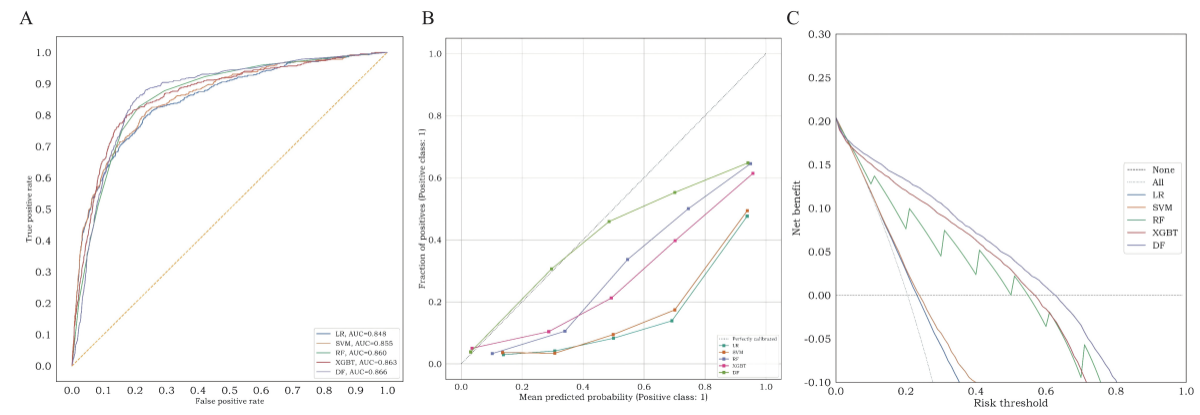
The performance of machine learning models in the external validation set. (A) area under receiver operating characteristic curve, (B) calibration curve, (C) decision curve analysis curve. AUC: area under the receiver operating characteristic curve; DF: deep forest; LR: logistic regression; RF: random forest; SVM: support vector machine; XGBT: extreme gradient boosting.

The data for the external validation set were obtained from Nanjing First Hospital and Sir Run Run Hospital affiliated with Nanjing Medical University, encompassing 3160 patients with CAP. The incidence of AKI was 20.4% (n=645) and the baseline clinical features are outlined in Table 1. Figure 5A presents the AUC of the 5 models in the external validation set. Among them, the DF model exhibited the most favorable predictive performance, achieving an AUC of 0.87 (95% CI 0.85-0.88), specificity of 0.93 (95% CI 0.93-0.94), and PPV of 0.63 (95% CI 0.59-0.67). The XGBoost model closely followed with an AUC of 0.86 (95% CI 0.85-0.88), accuracy of 0.84 (95% CI 0.83-0.85), and *F*_1_-score of 0.65 (95% CI 0.63-0.68). Detailed performance metrics are presented in Table 4. Calibration curves of the prediction models are depicted in Figure 5B. The results demonstrated that the DF model exhibited the best calibration ability in the external validation set. Figure 5C presents the decision curve analysis curves of the 5 models, indicating that the DF, XGBoost, and RF models yielded net benefits within the threshold range of 0-0.6. In the external validation set, the DF model showcased the highest predictive ability for AKI in patients with CAP.

**Table 4:** Detailed prediction performance of 5 machine learning models in external validation sets.

Models	AUC^a^, (95% CI)	Accuracy, (95% CI)	Sensitivity, (95% CI)	Specificity, (95% CI)	PPV^b^, (95% CI)	NPV^c^, (95% CI)	*F*_1_-score, (95% CI)	ECE^d^
LR^e^	0.85 (0.83-0.86)	0.45 (0.43-0.46)	0.97 (0.95-0.98)	0.32 (0.30-0.33)	0.27 (0.25-0.28)	0.97 (0.96-0.98)	0.42 (0.40-0.44)	0.38
SVM^f^	0.86 (0.84-0.87)	0.51 (0.49-0.52)	0.95 (0.94-0.96)	0.39 (0.38-0.41)	0.29 (0.27-0.30)	0.97 (0.96-0.98)	0.44 (0.42-0.46)	0.36
RF^g^	0.86 (0.85-0.87)	0.83 (0.82-0.84)	0.65 (0.62-0.68)	0.88 (0.87-0.90)	0.58 (0.55-0.61)	0.91 (0.90-0.92)	0.61 (0.59-0.64)	0.16
XGBoost^h^	0.86 (0.85-0.88)	0.84 (0.83-0.85)	0.73 (0.70-0.76)	0.87 (0.86-0.88)	0.59 (0.56-0.62)	0.93 (0.92-0.94)	0.65 (0.63-0.68)	0.13
DF^i^	0.87 (0.85-0.88)	0.83 (0.82-0.84)	0.44 (0.41-0.47)	0.93 (0.93-0.94)	0.63 (0.59-0.67)	0.87 (0.86-0.88)	0.52 (0.49-0.55)	0.05

^a^AUC: area under the receiver operating characteristic curve.

^b^PPV: positive predictive value.

^c^NPV: negative predictive value.

^d^ECE: expected calibration error.

^e^LR: logistic regression.

^f^SVM: support vector machine.

^g^RF: random forest.

^h^XGBoost: extreme gradient boosting.

^i^DF: deep forest.

### SHAP Values Evaluate Feature Importance

**Figure 6:**
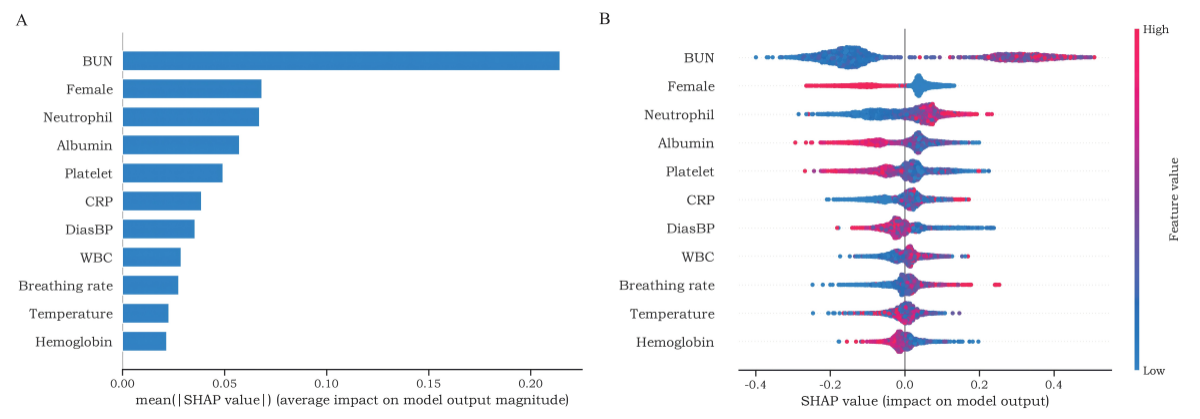
Shapley additive explanations (SHAP) interpretation and visualization of the deep forest model. (A) SHAP analysis shows a vital ranking of 11 features, and (B) SHAP analysis shows point estimation of features to model output. Each point represents a single patient in the data set. BUN: blood urea nitrogen; CRP: C-reactive protein; DiasBP: diastolic blood pressure; WBC: white blood cells.

We used SHAP to determine the importance ranking of the 11 features, as shown in Figure 6A. The significance of SHAP features is depicted in Figure 6B, where the x-axis represents the positive or negative contribution of a single feature to the model. The y-axis displays the 11 features in descending order of importance based on the absolute value of SHAP. A higher absolute value of SHAP indicates a greater contribution of the feature to AKI risk. The results revealed that BUN, neutrophil count, CRP, white blood cell count, breathing rate, and temperature made positive contributions, implying that higher values increased the risk of AKI. Conversely, female sex, albumin, platelet count, diastolic blood pressure, and hemoglobin made negative contributions, indicating that higher values decreased the risk of AKI.

**Figure 7:**
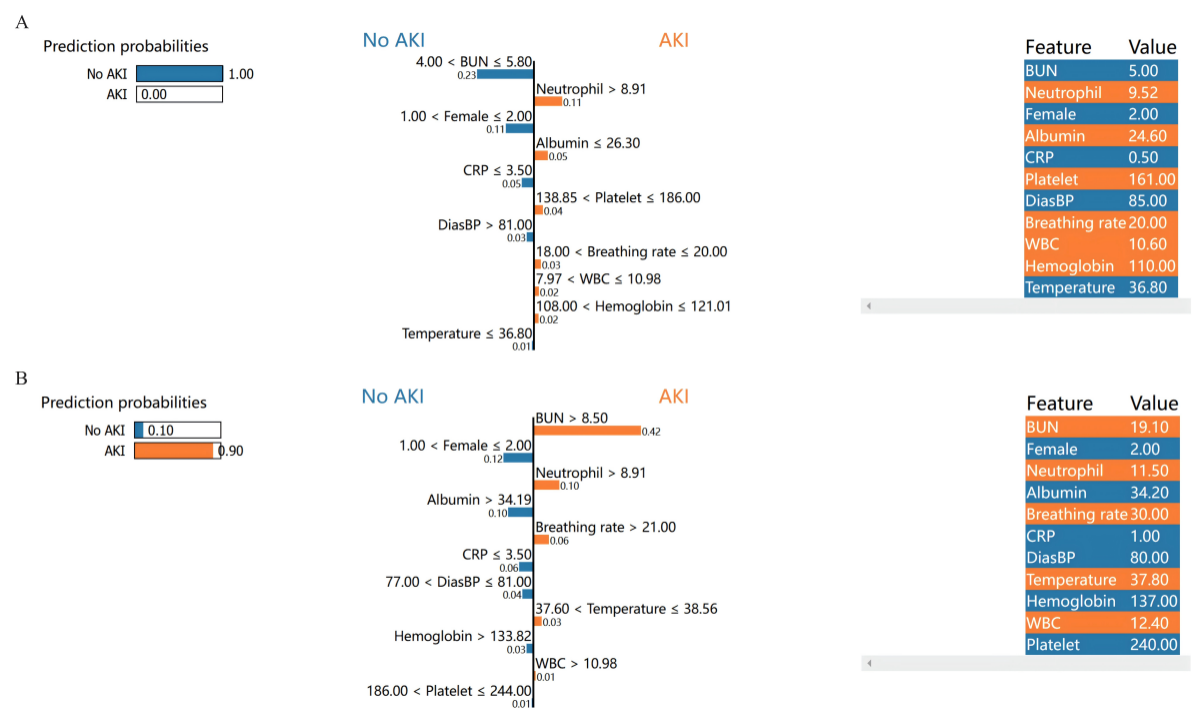
Local interpretable model-agnostic explanations (LIME) explaining individual’s prediction results. The left part of the figure shows predicted results using LIME. The middle part presents the impact of the 11 variables on acute kidney injury (AKI) or no AKI. The length of the bar for each feature indicates the importance (weight) of that feature in making the prediction. A longer bar indicates a feature that contributes more to (A) No AKI or (B) AKI. The right panel shows the critical values of these 11 variables when they had the greatest impact on (A) No AKI or (B) AKI. AKI: acute kidney injury; BUN: blood urea nitrogen; CRP: C-reactive protein; DiasBP: diastolic blood pressure; SHAP: SHapley Additive exPlanations; WBC: white blood cells.

In addition to explaining the overall contribution of the model using SHAP, we used LIME to individualize the contribution of the last 11 features in the prediction process. Figure 7A illustrates the predictive process for a patient without AKI. First, the 11 patient characteristics were ranked by importance. Blue indicates the contribution to the prediction as non-AKI, orange represents the contribution as AKI, and the corresponding prediction probability is displayed on the left. The specific values of the patient’s 11 features are presented on the right. In this case, the patient had a BUN level of 5.0 mmol/L, neutrophil count of 9.52×10^9^/L, breathing rate of 20 breaths/min, temperature of 36.8°C, white blood cell count of 10.6×10^9^/L, albumin level of 24.6 g/L, CRP level of 0.5 mg/L, diastolic blood pressure level of 85 mm Hg, hemoglobin level of 110 g/L, and platelet count of 161×10^9^/L. The probability of predicting non-AKI for this patient was 1.0.

Furthermore, Figure 7B demonstrates the predictive process for a patient with AKI. With a BUN level of 19.1 mmol/L, neutrophil count of 11.5×10^9^/L, breathing rate of 30 breaths/min, temperature of 37.8°C, white blood cell count of 12.4×10^9^/L, albumin level of 34.2 g/L, CRP level of 1 mg/L, diastolic blood pressure level of 80 mm Hg, hemoglobin level of 137 g/L, and platelet count of 240×10^9^/L, the probability of predicting AKI for this patient was 0.9.

### Establishment of a Web-Based Prediction Platform

**Figure 8:**
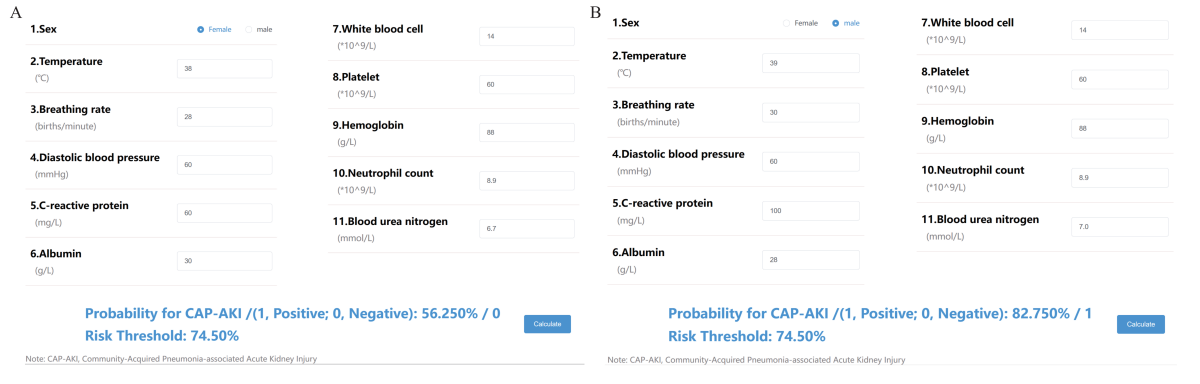
Web-based prediction platform of community-acquired pneumonia–associated acute kidney injury (CAP-AKI). (A) When 11 variables of a patient with community-acquired pneumonia were entered, the risk of CAP-AKI was calculated to be 56.25 %, and the result was 0, that is, the patient was not a high-risk group of CAP-AKI. (B) When 11 variables of a patient with community-acquired pneumonia were entered, the risk of CAP-AKI was calculated to be 82.75 %, and the result was 1, that is, the patient was a high-risk group of CAP-AKI. We set the risk threshold of our web calculator was 74.50% based on the best F1-score. When the predictive probability exceeds the risk threshold, the output is 1, otherwise the output is 0.

We developed a web-based prediction platform specifically designed for predicting AKI in patients with CAP (Figure 8). This platform uses the 11 selected variables as inputs to generate a risk value for predicting CAP-AKI. If the predicted risk value exceeds the predefined threshold of 74.5% set by the system, the result is considered positive, indicating that the patient falls into the high-risk category for developing CAP-AKI.

## Discussion

### Principal Findings

In this study, we used ML algorithms to develop an innovative prediction tool for CAP-AKI. CAP stands as a prevalent infectious disease globally, entailing considerable morbidity and mortality [11-13]. Among the array of complications linked with CAP, AKI has surfaced as a notable concern [14,15]. Our study unveils a noteworthy incidence of 15.8% for AKI among patients with CAP, indicating a substantial incidence of AKI within this population. Pinpointing the risk factors associated with AKI and crafting a predictive model are pivotal endeavors, enabling effective risk stratification and timely intervention for AKI. In this study, we identified the top 11 important predictors, including BUN, neutrophil count, respiratory rate, CRP, temperature, albumin, diastolic blood pressure, hemoglobin, platelet count, white blood cell count, and sex, as these variables can be easily collected in medical practice. In addition, our DF model showed good performance in both internal (AUC 0.89, 95% CI 0.84-0.92) and external (AUC 0.87, 95% CI 0.85-0.88) validation sets, indicating strong generalization ability.

ML techniques offer a promising path by harnessing the extensive pool of clinical data to offer insightful early-stage predictions of AKI. This approach potentially eliminates the necessity for additional AKI biomarker testing [16,17]. Hsu et al [18] constructed an AKI prediction model using 10 preprocedural indicators through an XGBoost algorithm, revealing an AUC of 0.767 on derivation and 0.761 on validation for any stage of AKI. Other researchers also found that XGBoost [19] and recurrent neural network [20] could perform well in predicting cardiac surgery–associated AKI and in-hospital AKI. Due to the distinct etiology of AKI in patients with CAP compared with other causes such as cardiac surgery–related AKI or sepsis-induced AKI, there are differences in the characteristics and factors required for model establishment. Some unique features need to be incorporated, including respiratory status, body temperature, blood pressure, and inflammatory markers, which have a significant impact on the occurrence of AKI. These parameters may differ from those in AKI models related to other etiologies. This aspect has been further elaborated in the discussion section.

In this study, we used ML algorithms to develop an innovative prediction tool for CAP-AKI. Furthermore, we determined the top 11 important predictors in the DF model as prediction model variables as these variables can be collected easily in medical activities. These features comprehensively consider the patient’s basic characteristics (such as age and sex), disease status (such as blood pressure and Scr), and potential causes (such as infections and chronic diseases), providing a well-rounded informational foundation for the model from multiple perspectives. Similarly, AUC for AKI in patients with CAP of the internal validation and external validation cohorts was shown to be 0.86 (95% CI 0.84-0.92) and 0.87 (95% CI 0.85-0.88), respectively. In addition, we constructed SHAP to provide personalized interpretation for each patient. Subsequently, an online web risk calculator model for AKI in patients with CAP was established to predict the occurrence of AKI within 7 days upon patients’ hospital admission. The median time for diagnosing AKI in our study was the third day. Although there was a certain time difference between the diagnosis time of AKI and the time of the indicators we included, our results showed that the model still exhibited good predictive performance in the validation set, and we believed that this time difference could be used as the best time for early intervention in high-risk patients who may develop AKI.

Among the 5 ML models used in this study, LR is commonly used in clinical settings. However, its performance is suboptimal when dealing with large-sample and high-dimensional data. RF exhibits good generalization ability and can prevent overfitting. XGBoost derived from RF is unaffected by multicollinearity and possesses characteristics such as flexibility and efficiency. DF is a novel ensemble-based method proposed in recent years, serving as an alternative to deep neural networks. It combines various ensemble-based methods with indivisible modules. Compared with deep neural network, DF has the advantage of achieving the optimal model with a small amount of training data, without the need for backpropagation, and has low computational costs. Zhang et al [21] found that the DF model achieved an AUC of 0.881 for predicting AKI after cardiac surgery, outperforming other ML models, including LR, XGBoost, and RF. Since its proposal, DF has been widely applied and has demonstrated good predictive performance in various fields such as radiological diagnosis of COVID-19 and emotion recognition [22,23].

In this study, we conducted a ranking analysis of the included feature variables and observed that several factors, namely BUN, neutrophil count, respiratory rate, CRP, temperature, albumin, diastolic blood pressure, hemoglobin, platelet count, white blood cell count, and sex, exerted a significant impact on the final model. Among these features, BUN emerged as the most crucial indicator for AKI. Similar to Scr, BUN serves as a reflection of renal function and carries substantial predictive weight for AKI occurrence. These findings align with the diagnostic criteria recommended by the KDIGO and are consistent with previous clinical investigations. Elevated levels of white blood cells, neutrophils, and CRP are also risk factors for AKI in patients. AKI is closely associated with renal and systemic inflammation. In the presence of infection, inflammatory factors play a critical role in combating microbial pathogens and facilitating tissue repair. White blood cells, including neutrophils, can infiltrate the injured kidneys through the circulatory system, triggering the production of cytokines, chemokines, and other inflammatory mediators, ultimately leading to renal damage [24]. CRP, as a plasma protein synthesized in the liver, serves as a nonspecific marker for systemic inflammatory response during the acute phase and is closely related to the severity of patients with CAP. Consistent with the findings of Wang et al [25] in patients with AKI after myocardial infarction, we also found that elevated CRP levels were a risk factor for AKI in the cohort of patients with CAP. Hypoalbuminemia is another risk factor for AKI in patients with CAP. Animal experiments have shown that albumin can counteract the decline in arterial vascular reactivity caused by endotoxemia, alleviate ischemia-reperfusion injury, and possess anti-inflammatory effects. Therefore, reduced albumin levels significantly reduce a patient’s resistance to infection and stress damage [26]. Similar conclusions have been reached by Bang et al [27]. The relationship between hemoglobin levels and AKI has been widely studied, with anemia being an independent risk factor for AKI [28-30]. The findings of our study provide compelling evidence that platelet levels act as a protective factor for AKI in patients with CAP. These results align with the previous investigations conducted by Zhang et al [31], thereby reinforcing the notion that a decreased platelet count is significantly associated with an elevated risk of AKI occurrence.

The prediction process of ML algorithms can be likened to a “black box” [32], where the inner workings may not be readily understandable to clinical physicians. Therefore, it is crucial for physicians to not solely rely on predictive models but also use their expertise in making appropriate diagnostic and therapeutic decisions. In this study, we used SHAP and LIME techniques to visualize the overall and individual contributions of each indicator to the model’s predictive performance, providing additional insights to clinical physicians. By quantifying the individual risks of AKI occurrence through a web-based prediction platform, we enhanced the clinical utility of the predictive model and demonstrated its significant potential in AKI risk prediction.

In this study, a comprehensive model was developed to predict the occurrence of AKI in patients with CAP by integrating multiple feature variables, resulting in robust predictive performance in both internal and external datasets. In addition, to enhance the interpretability of the best-performing DF model, visual explanations were provided using SHAP and LIME techniques.

Furthermore, a web-based prediction platform was established to augment the clinical utility of the predictive model, offering practical applications in real-world health care settings. The threshold of 74.5% was chosen based on the method of selecting the maximum *F*_1_-score on the training set. In a real-world clinical setting, a model with low sensitivity means that a significant number of patients with AKI might not be identified. This could lead to missed opportunities for early intervention and treatment, potentially worsening patient outcomes. However, the higher PPV suggests that when the model does predict AKI, it is more likely to be correct. This reduces the burden of false positives on clinicians, who might otherwise need to follow up on many non-AKI cases, saving time and resources.

The choice of threshold reflects a preference for higher precision, which might be suitable in situations where the cost of false positives is high, such as in scenarios with limited resources or when unnecessary treatments pose risks to patients. However, the trade-off with lower sensitivity means clinicians must be aware that not all cases of AKI will be caught by the model, and they should continue to use their clinical judgment and other diagnostic tools to ensure comprehensive patient care. Remarkably, the DF model outperformed others in both the internal and external validation cohorts.

However, it is important to acknowledge the limitations of this study. First, it should be noted that this was a retrospective study, which may have introduced certain limitations in terms of the completeness, accuracy, and homogeneity of data recording compared with prospective studies. In addition, the presence of selection bias cannot be ruled out, highlighting the need for further validation through prospective research. Second, the collection of urine output data posed challenges, resulting in cases of incomplete data collection. Furthermore, the consideration of urine output standards was not incorporated in this study, which could have provided additional valuable insights. Finally, while the constructed risk prediction model shows promise, it remains uncertain whether its implementation in clinical practice would translate into tangible benefits for patients. Therefore, prospective and multicenter studies are necessary to comprehensively evaluate the effectiveness and real-world impact of the model. Acknowledging and addressing these limitations will be essential in further refining the understanding and application of the findings in future research.

In summary, this study presents a valuable early prediction tool utilizing ML techniques to identify patients with high risk of AKI among patients with CAP. The developed predictive model has the potential to aid physicians in the early identification of patients at a higher risk of AKI during their hospitalization, enabling prioritized treatment interventions and ultimately improving patient outcomes. Moving forward, our future research endeavors will focus on prospective evaluation of the effectiveness of our AKI prediction model and determining its impact on the prognosis of patients with AKI in real-world clinical practice. In addition, the model could be applied to other etiologies of AKI, such as sepsis-induced AKI, to assess its generalizability.

### Conclusion

This study developed a ML-based prediction model for CAP-AKI, demonstrating excellent predictive performance in both internal and external validation cohorts. By integrating easily accessible clinical variables, the DF model achieved high accuracy and interpretability, offering robust support for early risk assessment and targeted intervention in patients with CAP-AKI. In the future, we hope to reduce the risk of CAP-AKI through our online prediction platform.

## Appendix

Multimedia Appendix 1 Detailed description of the machine learning methods.

## Abbreviations

AKI: acute kidney injury
ALT: alanine aminotransferase
AST: aspartate aminotransferase
AUC: area under the receiver operating characteristic curve
BUN: blood urea nitrogen
CAP: community-acquired pneumonia
CAP-AKI: community-acquired pneumonia–associated acute kidney injury
CKD: chronic kidney disease
CKD-EPI: Chronic Kidney Disease Epidemiology Collaboration
CRP: C-reactive protein
DF: deep forest
ECE: expected calibration error
eGFR: estimated glomerular filtration rate
KDIGO: Kidney Disease Improving Global Outcomes
LIME: local interpretable model-agnostic explanation
LR: logistic regression
MIMIC-III: The Medical Information Mart for Intensive Care III
ML: machine learning
OOB: out-of-bag
PPV: positive predictive value
RAAS: renin-angiotensin-aldosterone system
RF: random forest
Scr: serum creatinine
SHAP: Shapley additive explanation
SMOTE: synthetic minority over-sampling technique
VIF: variance inflation factor
XGBoost: extreme gradient boosting
