# Supplementary material for: A Machine Learning–Based Prediction Model for Acute Kidney Injury in Patients With Community-Acquired Pneumonia: Multicenter Validation Study
Source: J Med Internet Res. 2024 Dec 19;26:e51255. doi: 10.2196/51255 (PMC11695953; doi:10.2196/51255)
Supplement: Multimedia Appendix 1 [file jmir_v26i1e51255_app1.docx]

### Supplementary Material.

LR: The LR algorithm is widely employed in epidemiology and medical statistics as a popular modeling method for binary outcomes. It falls under the category of generalized linear models and is a supervised statistical learning approach. LR is commonly used in medical research for tasks such as screening disease risk factors, predicting disease occurrence, and estimating the probability of developing a specific ailment. The LR algorithm assumes a specific data distribution and utilizes maximum likelihood estimation to estimate its parameters. One of its advantages is that it generates regression coefficients and statistics based on hypothesis testing, making the results easily interpretable. However, LR requires prior selection of relevant covariates for modeling and the consideration of multicollinearity among covariates. Although it has a simple form, fitting it to the appropriate data distribution can be challenging.

SVM: SVM is a widely used classification algorithm in ML and one of the most influential algorithms in supervised learning. The core concept of SVM is to establish an optimal decision hyperplane that maximizes the distance between the closest samples of different classes on both sides of the hyperplane. This approach provides good generalization capabilities for classification problems. For multidimensional sample sets, SVM randomly generates a hyperplane and iteratively classifies samples until the sample points from different categories are located on opposite sides of the hyperplane. There may be multiple hyperplanes satisfying this condition, and SVM aims to find the hyperplane that maximizes the classification accuracy while maximizing the margin area on both sides of the hyperplane for optimal linear separable sample classification. We developed the SVM models using a linear basis function kernel with regularization parameter C set to 1.0.

RF: RF is a robust ensemble learning method based on Bagging that can be applied to classification, regression, and other problems. It improves upon the decision tree algorithm by combining multiple decision trees. Each tree in the forest is built using an independent sample, and all trees in the forest share the same distribution. The classification error depends on the classification abilities of individual trees and the correlations between them. RF employs a random feature selection method to split nodes and compares the errors generated under different circumstances to select features. The number of selected features is determined based on intrinsic estimation error, classification ability, and detectable correlations. While the classification ability of an individual tree may be limited, by generating a large number of decision trees, RF can select the most likely classification for a test sample by aggregating the results of each tree. The algorithm was deployed in our data set with 10 trees in the forest using the Gini criterion for splitting, and the tree nodes are expandeduntil all leaves contain less than 2.

XGBoost: XGBoost is an improved algorithm based on gradient boosting decision trees (GBDT). It incorporates three main enhancements: 1) Second-order Taylor expansion of the loss function, utilizing first-order and second-order derivative information; 2) Introduction of a regularization term in the loss function to control model complexity; 3) Feature sampling inspired by the RF algorithm. XGBoost also belongs to the ensemble learning approach. Unlike the RF algorithm, its learners (e.g., decision trees) exhibit strong dependencies, where each base learner is built based on the previous generation of base learners (boosting algorithm). Ultimately, all weak learners are combined into a strong learner. The algorithm was deployed in our data set with a learning rate of 1.0, estimators of 100 and the tree nodes are expanded until all leaves are pure or until all leaves contain less than 2.

DF: DF is a supervised ensemble learning algorithm inspired by deep learning theory and deep neural networks. Its full name is multi-Grained Cascade Forest (GCForest), proposed by Professor Zhou Zhihua of Nanjing University in 2017 as an alternative to deep neural networks (DNN). The algorithm integrates forests composed of trees and connects them in series to facilitate feature learning and enhance classification accuracy. Compared to DNN, DF offers easier parameter adjustment and lower computational overhead. The model can handle data of different sizes, and its complexity can be adaptively scaled. The generation of each cascade utilizes cross-validation to prevent overfitting. The DF algorithm consists of two stages: Multi-Grained Scanning and Cascade. The algorithm was deployed with 100 trees in the forest and the tree nodes are expanded until all leaves contain less than 2 ,max count of layers was set to 20 to avoid overfitting.
